# Interplay between the Gut Microbiota and Inflammatory Mediators in the Development of Colorectal Cancer

**DOI:** 10.3390/cancers13040734

**Published:** 2021-02-10

**Authors:** Gwangbeom Heo, Yunna Lee, Eunok Im

**Affiliations:** College of Pharmacy, Pusan National University, Busan 46241, Korea; gbheo@pusan.ac.kr (G.H.); yunnalee@pusan.ac.kr (Y.L.)

**Keywords:** microbiota, colorectal cancer, inflammatory mediators, fecal microbiota transplantation, germ-free animal

## Abstract

**Simple Summary:**

The development of colorectal cancer (CRC) can be affected by various inflammatory mediators, such as tumor necrosis factor, nuclear factor kappa B, interleukins, and interferons. Moreover, these inflammatory mediators can be reciprocally affected by gut microbiota. This review demonstrates the correlation of gut microbiota, inflammatory mediators, and CRC by summarizing studies with germ-free animals, antibiotic-treated animals, fecal microbiota transplantation, administration of specific microbiota, transgenic mice, and experimental models of CRC. It is clear that gut microbiota affect CRC through inflammatory mediators, though whether they promote or inhibit CRC depends on the context. Therefore, modulation of gut microbiota can be a good strategy for CRC prevention and control or be adjunctive therapy for CRC.

**Abstract:**

Inflammatory mediators modulate inflammatory pathways during the development of colorectal cancer. Inflammatory mediators secreted by both immune and tumor cells can influence carcinogenesis, progression, and tumor metastasis. The gut microbiota, which colonize the entire intestinal tract, especially the colon, are closely linked to colorectal cancer through an association with inflammatory mediators such as tumor necrosis factor, nuclear factor kappa B, interleukins, and interferons. This association may be a potential therapeutic target, since therapeutic interventions targeting the gut microbiota have been actively investigated in both the laboratory and in clinics and include fecal microbiota transplantation and probiotics.

## 1. Introduction

Colorectal cancer (CRC) is the third most frequent type of cancer and the second leading cause of cancer-related death worldwide [1]. About 80% of CRC cases are sporadic, and hereditary and colitis-associated CRC account for the remaining cases [2]. Various types of immune cells participate in carcinogenesis and the development of CRC by producing inflammatory mediators or triggering other cells to produce the mediators. Not only innate immune cells such as macrophages, dendritic cells (DCs), and natural killer (NK) cells but also adaptive immune cells play important roles in the tumor microenvironment. There are tumor-infiltrating immune cells including macrophages, neutrophils, and mast cells in the tumor. Macrophages produce IL-23 and this can induce release of the inflammatory cytokines such as interleukin-1 (IL-1), IL-6, IL-8, and tumor necrosis factor-α (TNF-α) from other cells including stromal, epithelial, and endothelial cells [3]. In addition, macrophages in tumor microenvironment, so-called tumor-associated macrophages, can promote angiogenesis and metastasis by secreting vascular endothelial growth factor (VEGF) in CRC [4]. On the other hand, T cells and their cytokines are involved in the development of cancer. CD8^+^ T cells can produce interferon-γ (IFN-γ), a well-known potent tumor-inhibiting cytokine and CD4^+^ Th1 cells can produce IFN-γ and IL-4 [5]. The inflammatory mediators also affect tight junction, which is an important factor that maintains intestinal epithelial barrier integrity. Some studies reported the positive effects of IL-6, IL-10, and IL-17 on intestinal epithelial integrity [6,7,8,9].

Microbiota include bacteria, fungi, viruses, archaea, and protozoans. Microbiota exist throughout the body, including in the gastrointestinal, respiratory, and genital tracts [10,11,12]. Studies have investigated the role of microbiota in the development of CRC. Both tumor-promoting and -inhibiting effects of microbiota have been reported. Some studies have indicated the involvement of inflammatory mediators in the link between microbiota and CRC development [13,14,15]. This review focuses on the interaction between the gut microbiota and inflammatory mediators in the development of CRC.

## 2. Microbiota, Inflammatory Mediators and CRC

The association between inflammatory mediators and CRC has been reported over the past decades. Numerous inflammatory mediators such as TNF-α, IL-6, IL-10, IL-23, and transforming growth factor-β (TGF-β) were found to be associated with carcinogenesis and the development of CRC and their effects include pro-tumor, anti-tumor, and double-edge effects in CRC pathogenesis [16,17].

The microbiota can influence the host directly and indirectly. Studies have reported a correlation between the gut microbiota and CRC. Pathways related to inflammatory mediators play a pivotal role in this process. Understanding which inflammatory mediators are affected by the gut microbiota and how these are affected enables the mediators to be modulated via the microbiota, rather than via anti-inflammatory agents. Indeed, as the roles of the gut microbiota in various diseases have been gradually elucidated, the manipulation of the gut microbiota has become a suitable therapeutic target. In recent decades, researchers and healthcare professionals have attempted to control or adjust the composition of the gut microbiota. Fecal microbiota transplantation (FMT) and probiotics are two major ways of manipulating the gut microbiota [18,19,20]. Thus, these provide alternative options for the treatment of patients with inflammatory diseases and for inflammation-targeted approaches to cancer.

### 2.1. Tumor Necrosis Factor

TNF-α plays a pivotal role in inflammation and has been called a “master regulator” of inflammatory cytokine production [21]. Therefore, anti-TNF therapy has been widely used for the treatment of various inflammatory diseases [22,23,24].

In a clinical study, anti-TNF treatment with adalimumab (ADA) induced compositional changes in the microbiota of patients with Crohn’s disease (CD). The relative abundance of *Escherichia coli* was significantly reduced following 3-months’ treatment with ADA compared to that at baseline [25]. Borruel et al. cocultured the mucosa from ileal specimens obtained from CD patients with bacteria. Coculture of *Lactobacillus casei* and *L*. *bulgaricus* with the inflamed mucosa revealed significantly reduced TNF-α secretion in the supernatant compared to that in supernatant obtained from mucosa culture alone [26]. The correlation between the microbiota, inflammation, and TNF has been further investigated in animal models. For example, FMT inhibited the increased expression of *Tnf* in a mouse model of dextran sulfate sodium (DSS)-induced colitis [27].

In addition to inflammation, TNF is associated with carcinogenesis. TNF plays a dual role in cancer. It can induce the apoptosis of cancer cells by activating TNF receptor 1, a ubiquitously expressed receptor. Simultaneously, TNF can promote tumorigenesis and cancer progression by activating TNF receptor 2, which is expressed on immune cells and some cancer cells [28]. TNF-α expression is generally upregulated in patients with CRC. For example, TNF-α expression was found to be higher in patients with stage III and IV cancer than in those with stage I and stage II cancer [29,30]. In a model of azoxymethane (AOM)/DSS colitis-associated cancer, TNF receptor p55-deficient (TNF-Rp55KO) mice presented lower tumor incidence than wild-type (WT) mice. Mucosal infiltration of inflammatory cells such as neutrophils and macrophages was also lower in the TNF-Rp55KO mice than in the WT mice [31].

The correlation between TNF, the microbiota, and CRC has been studied in various ways. Purified p75 and p40 proteins from *Lactobacillus rhamnosus* GG (LGG), a probiotic bacterium used in yogurt, demonstrated anti-inflammatory and anti-apoptotic effects in colon epithelial cells. p75 and p40 inhibited TNF-induced apoptosis in HT-29 human CRC cells and increased Akt, p38, and ERK1/2 MAPK activation. p75 and p40 also ameliorated TNF-induced epithelial damage in cultured mouse colon explants [32].

To investigate whether the gut microbiota influence inflammation in the tumor microenvironment and tumor immunotherapy, Iida et al. employed an antibiotic cocktail (ABX) comprising vancomycin, imipenem, and neomycin to deplete the gut microbiota. They investigated the effect of tumor immunotherapy using a combination of inhibitory IL-10 receptor antibodies (anti-IL-10R) and CpG-oligodeoxynucleotides (ODN), a ligand of toll-like receptor 9, in a mouse xenograft model of MC38 colon carcinoma. ABX treatment reduced the efficacy of anti-IL-10R/CpG-ODN immunotherapy in the xenograft model. Efficacy of the immunotherapy was also inhibited in *Tnf*^−/−^ mice; however, there was no synergism between ABX treatment and *Tnf* deficiency. *Tnf* expression was increased by anti-IL-10R/CpG-ODN therapy, and this elevation was inhibited by ABX treatment. These findings were confirmed in germ-free (GF) mice. ABX treatment also reduced TNF-positive cells and TNF secretion in monocytes, macrophages, and DCs. Oral administration of lipopolysaccharide (LPS) significantly restored the ABX-induced reduction of *Tnf* expression in tumors. This implies that the gut microbiota and LPS are involved in TNF production in the tumor microenvironment in response to immunotherapy [33].

Yang et al. aimed to identify a link between TNF, microbiota, and CRC. They assessed the effects of TNF blockade and genotoxin colibactin-producing (*clb*^+^) *E. coli* in a model of colitis-associated cancer using DSS/*Apc*^min/+^ mice and *Apc*^min/+^; *Il10*^−/−^ mice. In the DSS/*Apc*^min/+^ mouse model, *Apc*^min/+^ mice were pre-treated with ABX containing streptomycin, bacitracin, gentamycin, and ciprofloxacin and colonized with *E. coli* K12 (control), NC101 (*clb*^+^), or NC101Δ*clbP*. The mice were then supplied with DSS in drinking water for 7 days. Tumor numbers were significantly higher in the NC101-colonized mice than in the K12 or NC101Δ*clbP*-colonized mice. In addition to the DSS/*Apc*^min/+^ mouse model, NC101 also promoted tumorigenesis compared to K12 and NC101Δ*clbP* in the GF *Apc*^min/+^; *Il10*^−/−^ mice. In both the ABX-treated DSS/*Apc*^min/+^ mice and GF *Apc*^min/+^; *Il10*^−/−^ mice colonized with NC101, TNF blockade with a TNF monoclonal antibody significantly inhibited tumor development. Interestingly, tumor-inhibiting effect of TNF therapy in NC101-colonized mice was disappeared when the anti-TNF-treated mice were co-housed with PBS-treated mice in the DSS/*Apc*^min/+^ mouse model. TNF blockade also influenced the microbiota composition and activity. In addition, the GF *Apc^min^*^/+^ mice received cecal contents from phosphate-buffered saline (PBS)-treated or anti-TNF-treated mice and were then supplied with DSS. The recipients of cecal contents from the anti-TNF-treated mice revealed significantly lower tumor numbers than the recipients of cecal contents from the PBS-treated mice [34]. According to a previous study, colibactin from *E. coli* induced DNA damage in colon epithelial cells [35]. Collectively, these data indicate that colibactin from *E. coli* promotes tumor development in a TNF-related manner. Similarly, TNF plays a crucial role in carcinogenesis in a microbiota-dependent manner.

In contrast to its role in inflammation, in cancer, TNF deficiency decreases the susceptibility of tumors to immunotherapy, whereas TNF blockade reduces colorectal carcinogenesis. This provides further opportunities for future research.

### 2.2. Nuclear Factor Kappa B

NF-κB is a pro-inflammatory transcription factor that induces various pro-inflammatory genes. The NF-κB signaling pathway can be activated by various stimuli such as microbial components, growth factors, and cytokines. NF-κB provokes defense mechanisms in the host; however, prolonged activation can induce tissue damage and carcinogenesis [36,37].

NF-κB is important in inflammation and in the initiation and progression of cancer [38,39]. NF-κB links inflammation to cancer via factors such as cyclins, Bcl-2 family members, IL-6, and cyclooxygenase-2 [40]. NF-κB can suppress the immune response by interfering in the immune surveillance of innate and adaptive immune cells [41]. The expression of NF-κB in tumor tissue from patients with CRC was significantly higher than that in adjacent normal tissue and that from patients without CRC [42].

*Fusobacterium nucleatum*, a Gram-negative anaerobe bacterium that is present in the oral cavity, was found to be associated with CRC in recent studies [43,44]. *F. nucleatum* showed tumor-promoting effects in a mouse xenograft model of HCT116 CRC cells and *Apc*^min/+^ mice. The tumor-promoting effect of *F. nucleatum* was lost in miR21a-deficient mice. To elucidate the underlying mechanism about the relationship between *F. nucleatum* and miR21, HCT116 CRC cells were treated with *F. nucleatum*. Yang et al. found *RASA1* as a target gene of miR21in miR21 overexpressed or miR21- deleted cells. Furthermore, in *F. nucleatum*-treated HCT116 cells, Toll-like receptor (TLR) 4 and MYD88 expression levels were up-regulated. NF-κB p65 and p50, a well-known downstream effector of TLR4 signaling, were also upregulated and IκBα, an inhibitor of NF-κB, was downregulated compared with that in vehicle-treated cells. Silencing NF-κB significantly suppressed the proliferation and migration of *F. nucleatum*-treated HCT116 cells. In addition, NF-κB activity was higher in CRC tissue with relatively high levels of *F. nucleatum* than in tissue with low levels of *F. nucleatum*. These results suggest that *F. nucleatum* regulates the TLR4/MYD88/NF-κB signaling pathway to promote tumor development [45].

*E. coli* has been shown to activate the NF-κB pathway in CRC cells. In *E. coli* SK3842-infected Caco-2 CRC cells, NF-κB activity was upregulated and IκBα was downregulated compared with that in control cells. In addition, pro-apoptotic Bcl2, BclXL, and survivin were upregulated in infected CRC cells, and cell migration was increased, suggesting the tumorigenic effects of *E. coli* SK3842 [46].

*Peptostreptococcus anaerobius*, a Gram-positive anaerobe bacterium, can promote CRC development via the NF-κB pathway. In a recent study by Long. et al., *Apc*^min/+^ mice were treated with ABX containing ampicillin, neomycin, metronidazole, and vancomycin and then colonized with *P. anaerobius*. Tumor numbers were significantly higher in *P. anaerobius*-colonized mice than in PBS-treated mice. The protein levels of NF-κB and phospho-NF-κB and the mRNA expression of *Il10* and *Infg* were increased in the tumor tissue of the *P. anaerobius*-colonized mice. These effects were inhibited following treatment with the integrin inhibitor RGDS peptide. The authors suggested that *P. anaerobius* modulated the integrin α_2_/β_1_-phosphoinositide 3-kinase-Akt-NF-κB signaling pathway [47].

Collectively, these data demonstrate that various microbiota and their metabolites can induce CRC development via the NF-κB signaling pathway.

### 2.3. Interleukin-1

IL-1 plays a key role in the inflammatory response and CRC development by modulating the innate and adaptive immune systems [48]. Notably, IL-1 secretion is enhanced in various inflammatory diseases [49,50].

Researchers have reported both the tumor-promoting and suppressive effects of the IL-1 pathway. Generally, IL-1 promotes tumorigenesis and tumor metastasis in CRC [51]. In colorectal specimens from patients with CRC, the expression of *Il1b* was higher in CRC tumors than in normal colorectal mucosa [52]. In addition, the tumor size was reduced following IL-1 receptor antagonist (IL-1Ra) treatment in xenograft models derived from WIDR human CRC cells [53]. In the AOM/DSS CRC mouse model, colon tissue from tumor-bearing mice showed significantly higher IL-1β production than that from naïve mice. IL-1Ra significantly reduced the number of tumors. Immunohistochemistry analysis revealed higher numbers of apoptotic cells in colon tissue from IL-1Ra-treated mice than in that from vehicle-treated mice. In addition, intestinal epithelial cells isolated from IL1-Ra-treated mice presented lower levels of phosphorylated NF-κB and higher levels of IκBα than those isolated from vehicle-treated mice [54].

In contrast, the tumor-suppressive effects of IL-1 have also been reported. Dmitrieva-Posocco et al. suggested cell-type-specific responses to IL-1 in CRC. The myeloid-specific deletion of IL-1R1 increased tumor multiplicity and size in an *APC* mouse model. However, IL-1R1 deletion in colonic epithelial cells reduced the tumor number. The tumor-promoting effect of IL-1R1 deletion was inhibited via ABX pre-treatment, suggesting a role for the microbiota in the tumorigenic effects of IL-1 [55]. Anakinra, an IL-1Ra drug approved for the treatment of rheumatoid arthritis, potentiated the efficacy of fluorouracil plus bevacizumab therapy in patients with metastatic CRC [56,57]. Other IL-1-targeting agents, including rilonacept, canakinumab, and gevokizumab, have been used clinically and studied in various inflammatory diseases and cancers [58].

Studies have reported a correlation between the gut microbiota and the IL-1 pathway. Unfortunately, no studies have directly linked IL-1, the microbiota, and CRC. However, this correlation can be assumed indirectly based on microbiota-IL-1 research and IL-1-CRC research. Seo et al. investigated the effects of the gut microbiota on IL-1β production following intestinal injury. GF mice had significantly lower levels of IL-1β in fecal contents than conventional mice. The levels of IL-1β in lamina propria (LP) cells from DSS-treated conventional mice were significantly higher than those in LP cells from vehicle-treated conventional mice. However, there was no significant difference in IL-1β production in LP cells from GF mice following DSS treatment. In addition, *Il1b*^−/−^ mice were protected against DSS-induced colitis and presented decreased body weight loss and disease activity index determined by stool consistency and hemoccult. These protective effects of *Il1b* deficiency were lost following antibiotic treatment [59]. GF mice presented significantly lower levels of IL-1β in the small intestine upon *Toxoplasma gondii* infection than control mice [60].

FMT, which involves the exogenous manipulation of the gut microbiota, inhibited the inflammation-induced increase in colonic *Il1b* expression in a mouse model of DSS-induced colitis [27]. VSL#3, a mixture of four *Lactobacillus* strains (*L*. *paracasei*, *L*. *plantarum*, *L*. *acidophilus*, and *L*. *delbrueckii* subsp. *bulgaricus*), three *Bifidobacterium* strains (*B*. *longum*, *B*. *breve*, and *B*. *infantis*), and one *Streptococcus* strain (*S*. *thermophilus*), was administered to patients with CD who had recently undergone ileocolonic resection. The mRNA expression of *Il8* and *Il1b* in colonic mucosal biopsies from patients in the VSL#3-treated group was significantly lower than that in biopsies from patients in the placebo-treated group [61]. Rogier et al. reported that the IL-1 pathway affects the diversity and richness of the gut microbiota. IL-1 receptor antagonist knockout mice (*IL1rn*^−/−^) presented lower microbiota diversity-related parameters, including operational taxonomic units (OTUs) and Shannon indices, than WT mice. Additionally, LP cells from the small intestine of *IL1rn*^−/−^ mice produced higher levels of IL-17 in the presence of phorbol myristate acetate and ionomycin than those from WT mice [62]. These findings suggest that the gut microbiota can modulate IL-1 production and IL-1-mediated inflammation and vice versa.

### 2.4. Interleukin-6

IL-6 is a prototypical pro-inflammatory cytokine produced by immune cells. IL-6 levels are elevated in numerous inflammatory diseases and are often used as a marker of inflammation. [63,64]. IL-6 is also an important molecular link between inflammation and cancer. Under inflammatory conditions, NF-κB-induced IL-6, secreted from immune cells or tumor cells, can lead to cancer progression and metastasis via the IL-6/signal transducer and activator of transcription 3 (STAT3) signaling pathway [65,66,67].

Many studies have confirmed that IL-6 levels are elevated in CRC [68]. IL-6 mRNA expression was higher in tumor tissues than in the normal colorectal mucosa in patients with CRC [52]. In a meta-analysis, Xu et al. showed that serum IL-6 could be used for the diagnosis of CRC and demonstrated an association between elevated serum IL-6 and poor prognosis. Their analysis showed that patients with high serum IL-6 levels had lower overall survival and disease-free survival than patients with low serum IL-6 [69]. The blockade of IL-6 signaling using an IL-6 receptor antibody reduced tumor development in an AOM/DSS mouse model [70]. Thus, IL-6-targeting therapies such as siltuximab and tocilizumab have been developed, and the anticancer effects of these drugs have been elucidated [71].

Some studies have investigated the role of the microbiota and IL-6 in intestinal diseases, including CRC. Wang et al. investigated the effects of FMT in patients with ulcerative colitis. Fecal samples from healthy donors were administered to patients through colonoscopy. FMT was performed three times every 2–3 months, and after the second administration, IL-6 expression was found to be significantly lower in serum compared to that at baseline. The authors also found that 87.5% of patients demonstrated a clinical response to FMT [72]. Djaldetti et al. investigated the effects of six mixed microbial strains—*Streptococcus thermophilus*, *Lactobacillus rhamnosus*, *L*. *acidophilus*, *L*. *casei*, *Bifidobacterium bifidum*, and *B*. *longum*—on immune cells in vitro. Peripheral blood mononuclear cells (PBMCs) were treated with the mixture; then, cytokine levels in the PMBC supernatants were evaluated using an enzyme-linked immunosorbent assay. It was found that LPS-induced IL-6 secretion was inhibited in the group treated with the bacterial mixture compared to that in the control [73]. ABX treatment upregulated the serum concentration and macrophage expression of IL-6 compared to the values in control mice with HT29 CRC xenografts [74]. Burrello et al. evaluated the effects of FMT in chronic intestinal colitis induced via three cycles of 7-day DSS treatment. Colonic expression of *Il6* was lower in FMT-treated mice than in FMT-untreated mice [75]. The microbiota metabolite butyrate inhibited LPS-induced IL-6 secretion in colon LP macrophages in vitro. Butyrate also inhibited *Il6* and *Nos2* mRNA expression in colon LP macrophages [76,77]. *F. nucleatum*, which has tumor-promoting and NF-κB-activating effects, is also associated with IL-6 in CRC. Chen et al. reported that IL-6/STAT3/c-Myc signaling was upregulated in patients with *F. nucleatum*-positive CRC compared to that in those with *F. nucleatum*-negative CRC [78]. In summary, evidence suggests that the immune-modulating effects of the gut microbiota are strongly related to IL-6. As IL-6 is a key player in the inflammatory pathway of CRC, the microbiota may also participate in the pathway in association with IL-6.

### 2.5. Interleukin-10

IL-10 is an anti-inflammatory cytokine that suppresses immune cells such as T cells, NK cells, and macrophages [79,80]. Owing to the close association of IL-10 with inflammation, IL-10-deficient mice, which can develop spontaneous colitis, have been widely used as experimental colitis models [81,82].

Although IL-10 is generally considered an immunosuppressive cytokine, its role in cancer is controversial. IL-10 can exert both tumor-promoting and -suppressive effects [83,84,85]. The pathway downstream of IL-10 includes Janus kinase and STAT, indicating that IL-10 promotes cancer progression and metastasis in the same way as IL-6. STAT3, which is activated by the IL-10 receptor, can promote cell proliferation and survival in association with cyclin D1, cMyc, BclXl, Mcl1, and p53. STAT3 also facilitates cell invasion and migration by regulating matrix metallopeptidase-2 (MMP-2), MMP-9, and MMP-7 and modulating Rho and Rac. Angiogenesis can also be regulated by STAT3 via the upregulation of VEGF and hypoxia-inducible factor 1-alpha [51,86]. IL-10 interferes with the antitumor activity of the immune system by inhibiting antigen presentation in both antigen presenting cells and tumor cells and inducing T cell anergy [87]. Tumor cells produce IL-10 and can therefore evade the immune system [88].

In contrast, evidence suggests that IL-10 can suppress cancer development and metastasis [83]. For example, IL-10 can exert antitumor effects by modulating immune cells [84]. Mumm et al. used three experimental cancer models to investigate the role of IL-10 in cancer: A 7,12-dimethylbenzanthracene-induced mouse skin cancer model, a breast cancer model with FVB^MMTV−rtHer2^ transgenic mice, and xenograft models with CT-26 mouse colon carcinoma cells, 4T1 mouse breast cancer cells, and CM3 human leukemia cells. In these animal studies, IL-10 overexpression and exogenous IL-10 suppressed tumor development. Conversely, tumor development was enhanced in IL-10-deficient mice. In that study, a positive correlation was found between CD8^+^ T cells in tumors and IL-10 administration or expression [89]. Clinically, pegylated IL-10, also known as pegilodecakin, demonstrated antitumor effects by activating and sustaining CD8^+^ T cells in patients with renal cell cancer [90,91]. NK cells are also involved in the tumor-suppressing effect of IL-10. IL-10 increased NK cell activity and inhibited tumor development and metastasis. The inhibitory effect was reduced via the suppression of NK cell activity [92,93].

These roles of IL-10 can be influenced via the manipulation of the gut microbiota. Burrello et al. reported that IL-10 expression in colonic lysates and IL-10 secretion from colonic CD4^+^ T cells were enhanced in FMT-treated mice [27]. Mishima et al. also confirmed that IL-10 secretion and *Il10* mRNA expression in colon tissue were significantly lower in GF mice than in control mice; they showed that these were recovered upon FMT. FMT in GF mice increased IL-10-producing T cells (CD25^−^ CD4^+^ CD3^+^) and B cells (B220^+^ CD19^+^), which were characterized from colon LP cells. The colonic mRNA expression of *Ifng* was also increased by FMT in GF mice. However, this effect was inhibited in *Il10*^−/−^ mice, suggesting that FMT-induced cytokine modulation is associated with IL-10 [94].

Wei et al. evaluated the therapeutic effects of FMT in a model of DSS-induced colitis. Feces prepared from healthy mice were transplanted into mice with colitis. Body weight recovered rapidly in the FMT-treated mice. These mice also presented a lower disease activity index score following DSS cessation than the FMT-untreated mice. Histological damage and inflammatory cell infiltration were lower in the colon tissues of the FMT-treated mice than in those of the FMT-untreated mice. Following DSS cessation, FMT restored the expression levels of IL-10 and TGF-β in the colon to the control levels [95]. To investigate the correlation between the microbiota, IL-10, and colitis-associated CRC, Arthur et al. employed *E. coli-* and AOM-treated *Il10*^−/−^ mice. In their study, *Il10*^−/−^ mice developed spontaneous colitis and presented feces with less OTUs and a lower Margalef richness index than the WT mice. This indicates that the colitis induced by IL-10 deficiency reduced gut microbiota diversity and richness. AOM injection did not influence the compositional differences between *Il10*^−/−^ mice and WT mice. *E. coli* NC101-mono-associated, AOM-treated *Il10*^−/−^ mice presented higher tumor multiplicity than *Enterococcus faecalis* mono-associated, AOM-treated *Il10*^−/−^ mice. The authors also investigated the role of colibactin, a genotoxin produced by *E. coli*, in CRC development. *E. coli* NC101Δpks, which does not produce colibactin, exerted lower tumor-promoting effects than *E. coli* NC101 in AOM-treated *Il10*^−/−^ mice [96].

Thus, IL-10 can modulate the development of CRC, and this process can be influenced by the gut microbiota. However, the exact effects on tumor progression, whether tumor-promoting or -suppressive, should be further investigated owing to the dual role of IL-10 in cancer development.

### 2.6. Interleukin-17

The IL-17 family is composed of IL-17A–F, which are pro-inflammatory cytokines mainly secreted by Th17 cells [97]. IL-17 induces immune responses with several inflammatory mediators, including IL-1, IL-6, IL-23, TNF, and NF-κB [98,99]. IL-17 expression is upregulated in autoimmune diseases and inflammatory diseases, including inflammatory bowel disease [100,101,102].

IL-17 is associated with all processes of tumorigenesis, including tumor formation, proliferation, and angiogenesis. IL-17 can promote tumor formation by triggering IL-6 secretion and subsequently activating the STAT3 pathway. IL-17 can also promote tumor proliferation via the MAPK/extracellular signal-regulated kinase pathway and angiogenesis by stimulating the production of VEGF. In addition to angiogenesis, IL-17 directly stimulates the migration of CRC cells, suggesting a role in the promotion of CRC metastasis and invasion [103,104,105,106].

In a *CDX2P*-*NLS Cre*;*Apc*^+/loxP^ (CPC;APC) mouse model of CRC, IL17A receptor (IL17RA)-deficient mice were found to have fewer and smaller tumors and increased numbers of apoptotic cells in colonic tumors. In addition, *Il17ra*^−/−^ mice demonstrated higher sensitivity to radiotherapy than *Il17ra*^+/−^ mice. Tumor tissue from *Il17ra*^−/−^ mice presented lower IL6 mRNA expression than that from *Il17ra*^+/−^ mice [107]. In a mouse xenograft model with CT26 murine colon cancer cells, IL-17A-overexpressing cells presented significantly higher tumor growth than mock vector-treated cells [108]. In a clinical study involving CRC patients, those in the IL-17 low group had a higher survival rate and lower microvessel density, a marker of angiogenesis, in tumor tissues than those in the IL-17 high group. This suggests that IL-17 may be a prognostic marker for CRC [109]. IL-17 has emerged as a therapeutic target for metastatic CRC (mCRC) through the inhibition of pathologic angiogenesis. Ibrahim et al. suggested the use of biologic agents targeting IL-17 and the IL17 receptor for patients with mCRC, including secukinumab, ixekizumab, and brodalumab. These agents have been studied and approved for use in patients with various inflammatory diseases such as psoriasis, psoriatic arthritis, and ankylosing spondylitis [105].

*Bacteroides fragilis*, an anaerobic Gram-negative bacterium, is a general commensal bacterium in the gastrointestinal tract [110]. Enterotoxigenic *B. fragilis* (ETBF) was found to be more abundant in patients with CRC than in healthy controls [111]. ETBF promoted the development of CRC in an IL-17-dependent manner. ETBF-colonized Min (APC^+/−^) mice were found to have higher tumor formation than sham-treated Min mice. STAT3 expression in colon tissue and IL-17-producing CD3^+^ CD4^+^ T lymphocytes in the colon LP was also increased in the ETBF-colonized mice. Blockade of IL-17 with an IL-17A-blocking antibody significantly reduced ETBF-induced colonic tumor formation in Min mice [112].

CD4^+^ T cells, isolated from the mesenteric lymph nodes of GF mice, produced lower levels of IL-17A when stimulated with TGF-β and IL-6 than those isolated from conventional mice. In addition, colonic LP lymphocytes from GF mice produced less IL-17A than those from conventional mice [113]. *Il17a* mRNA expression in the distal colon was significantly lower in GF mice than in specific-pathogen-free (SPF) mice [94]. Grivennikov et al. reported that IL-17A expression was upregulated in tumors compared to that in normal tissues in both human and mouse CRC. In *Apc*^F/F^ and *Cdx2-Cre* (CPC-Cre) mice, a spontaneous mouse CRC model, the expression of *Il17a* mRNA was lower in both tumor and normal tissues from ABX-treated mice than in tissue from control mice [114]. Interestingly, like the ABX-treated and GF mice, the colonic expression of *Il17* was also lower in FMT-treated mice than in FMT-untreated mice in DSS-induced colitis [75].

Collectively, the evidence indicates that dysbiosis of the gut microbiota can influence intestinal IL-17 production, which can modulate the host immune response. Consequently, the gut microbiota can affect the development of CRC in an IL-17-dependent manner. However, although the role of IL-17 in CRC is relatively clear, the exact pro- and anti-tumor effects of the gut microbiota acting via IL-17 are dependent on multiple factors.

### 2.7. Interferons

Interferons (IFNs) are signaling proteins secreted in response to microbial infection that activate the innate immune system. There are three types of IFNs: Type I, II, and III. In humans, type I IFNs include IFN-α, β, ε, κ, and ω and type II IFNs include IFN-γ. The imbalance and dysregulation of IFNs are highly related to various inflammatory diseases, though the roles of individual IFNs in inflammation remain controversial [115,116,117,118].

As IFNs play pivotal roles in the immune response, their role in cancer has been researched over the past decades [119]. Type I IFNs are produced by both immune and tumor cells and exert strong antitumor effects [120]. Clinically, the presence of type I IFNs in the tumor microenvironment implies specific immune cell infiltrates, which elicit a favorable response to chemotherapy and radiotherapy [121]. To obtain immune privilege, cancer cells express less type I IFN receptor chain IFNAR1. This was confirmed in both human patients with CRC and using the AOM/DSS mouse model [122]. Tumor number and size were reduced in *Ifnar1*^S426A^ (SA) mice, which are resistant to IFNAR1 ubiquitination and degradation, compared with the values in WT mice in the AOM/DSS model. This demonstrates that endogenous type I IFNs are important for tumor development in CRC [122]. Furthermore, blockade of type I IFNs and IFNAR1 abolished the anticancer activity of chemotherapy in various mouse xenograft models. Consistently, exogenous type I IFNs were found to potentiate the efficacy of chemotherapy [123].

Like type I IFNs, IFNγ, a type II IFN, exerts strong antitumor effects by regulating the immune response [124]. IFNγ is produced by immune cells and increases the activity of cytotoxic T lymphocytes and NK cells [125,126]. More specific to cancer, IFNγ induces cell cycle arrest and apoptosis of cancer cells and inhibits angiogenesis [127,128]. However, recently, the pro-tumorigenic effects of IFNγ have also been reported. Chronic exposure to IFNγ enhanced tumor growth in a mouse xenograft model with hepatoma and mammary adenocarcinoma cells [129]. IFNγ may exert its pro-tumorigenic effect via the Jak/STAT signaling pathway, downstream of the IFNγ receptor [130]. Therefore, owing to this paradoxical role of IFNγ, a cautious approach is required when applying it in anticancer therapy.

The production of IFNs in the host can be affected by the modulation of the gut microbiota. To investigate the effects of the gut microbiota on IFNs, Yitbarek et al. used ABX-treated chickens in a viral infection model. ABX containing vancomycin, neomycin, metronidazole, amphotericin B, and ampicillin was added to drinking water for 2 weeks. Then, the chickens were challenged with the avian influenza virus subtype H9N2. The ABX-treated chickens presented reduced populations of bacteria such as *Firmicutes*, *Bacteroidetes*, *Actinobacteria*, and γ-*proteobacteria* and the genus *Enterococcus,* in their cecal contents. The ABX-treated chickens were found to have lower IFN-α and IFN-β expression in the ileum and cecal tonsils following viral infection than the ABX-non-treated chickens [131].

Ichinohe et al. reported that ABX-treated mice failed to induce acquired immunity. Mice were treated with ABX containing ampicillin, vancomycin, neomycin, and metronidazole and challenged with A/PR8 influenza virus. ABX-treated mice demonstrated significantly lower antibody titers than the control mice. CD4^+^ and CD8^+^ T cells from ABX-treated mice produced significantly less IFN-γ in response to viral infection than control mice [132]. The role of the gut microbiota in IFN production was also elucidated using a parasite infection model. In GF mice, IFN-γ induction and IL-1β expression were significantly lower in the small intestine upon *Toxoplasma gondii* infection than in SPF mice [60]. Consistent with the GF mouse study, supplementation of the gut microbiota enhanced IFN production. Yogurt, a fermented food containing *Lactobacillus delbrueckii* subsp. *bulgaricus* and *Streptococcus thermophilus*, enhanced colonic IFN-γ expression in a mouse model of 1,2-dimethylhydrazine-induced CRC [133]. However, FMT inhibited colonic IFN-γ expression. Burrello et al. reported that the colonic expression of *Ifng* was lower in FMT-treated mice than in FMT-untreated mice in a colitis model induced via three cycles of DSS and water. In that study, FMT did not influence the body weight or histological score [75]. Thus, the gut microbiota can modulate the host immune response in an IFN-dependent manner. Collectively, the results suggest that the modulation of the gut microbiota can influence the development of CRC via the modification of IFN production.

## 3. Conclusions

**Figure 1:**
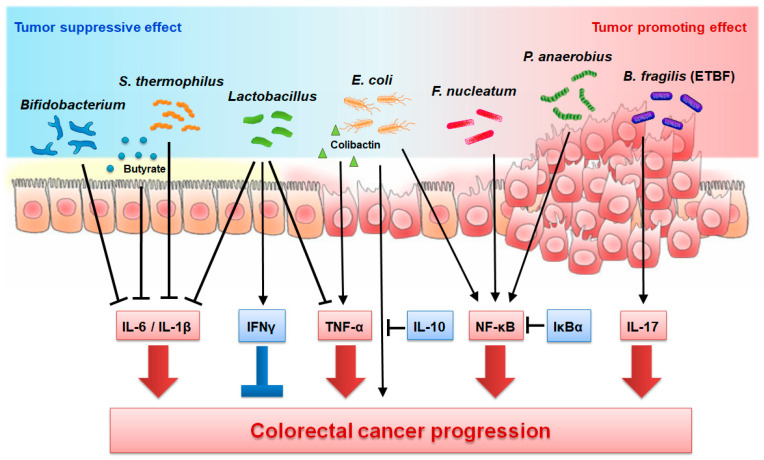
Roles of gut microbiota in regulation of inflammatory mediator production and colorectal cancer (CRC) progression. Some microbiota and their metabolites exert tumor suppressive effects by downregulating tumor promoting mediators such as IL-6, IL-1β, and TNF-α, and by upregulating the tumor suppressive mediator IFNγ. On the other hand, some microbiota and their metabolite exert tumor promoting effect by upregulating tumor promoting mediators such as TNF-α, NF-κB, and IL-17. arrow = promote; bar-headed line = inhibit.

**Figure 2:**
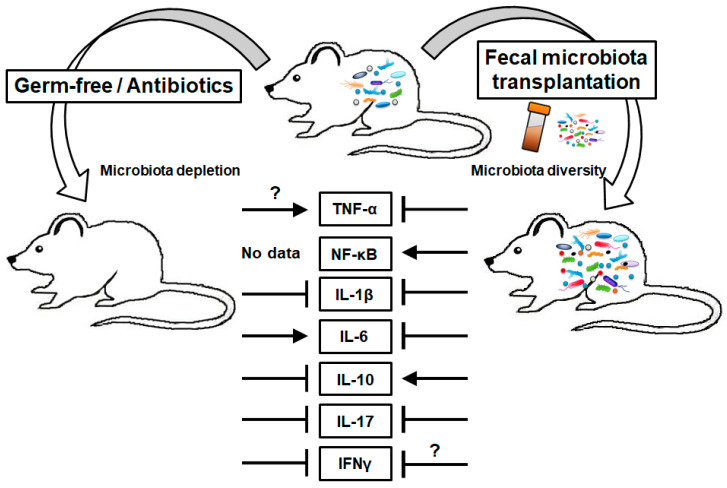
Effects of gut microbiota modulation on the production of inflammatory mediators. ?, controversial.

In this review, we discuss the correlation between the gut microbiota, inflammatory mediators, and the development of CRC. TNF, NF-κB, IL-1, IL-6, IL-10, IL-17, and IFNs have been shown to have a role in the development of CRC (Table 1). Numerous studies have demonstrated the role of the gut microbiota in CRC development. We attempted to connect the two aspects and present research about the direct link between the gut microbiota, inflammatory mediators, and CRC (Figure 1). In animal studies, researchers commonly use GF animals and CRC models to investigate the role of the gut microbiota in this condition. Mono-association with specific strains and the transplantation of large amounts of microbiota with FMT or probiotics have also been used (Figure 2). Genetically modified animals and inflammatory mediators or receptor-targeting antibodies have been used in further investigations. These studies have demonstrated that the gut microbiota affect CRC development. Whether the microbiota promote or inhibit tumor development depends on the strain and the products of the gut microbiota. Importantly, inflammatory mediators are involved in the development of CRC modulated by the gut microbiota. Future research should aim to determine the strain or microbial product that modulates tumor development and to elucidate the underlying mechanisms such as the inflammatory pathway involved.

## Figures and Tables

**Table 1:** Correlation between inflammatory mediators, CRC, and gut microbiota.

Mediators	Roles in CRC	Status in CRC	Status in GF or ABX-Treated Animal	Modulation by Exogenous Microbiota
TNF	Dual [28]	↑ [29,30]	↓ in tumor under immunotherapy [33] ↑ in serum [74]	↓ by FMT under DSS [27]
NF-κB	Promote [39]	↑ [42]	-	↑ by *P. anaerobius* [47]
IL-1	Dual [51,55]	↑, IL-1β [52]	↓ in LP under DSS [59] ↓ in SI under *T. gondii* infection [60]	↓ by FMT under DSS [27] ↓ by VSL#3 under CD [61]
IL-6	Promote [68]	↑ [52,68]	↑ in serum [74]	↓ by FMT under UC [72] ↓ by FMT under DSS [75] ↓ by mixture of 6 strains under LPS [73]
IL-10	Controversial [51]	↑ [134]	↓ in colon [94]	↑ by FMT [27,94,95]
IL-17	Promote [106,135]	↑ [114,135]	↓ in CD4^+^ T cell [113] ↓ in colon [94]	↓ by FMT under DSS [75]
IFNs	Suppress (α, β) [122] Controversial (γ) [124,129,130]	↓ IFNAR1 [122]	↓ α, β in ileum under viral infection [131] ↓ γ in T cells under viral infection [132] ↓ β, γ in SI under *T. gondii* infection [60]	↑ γ by *L. delbrueckii, S. thermophilus* under DMH-induced CRC [133] ↓ γ by FMT under DSS [75]

↑, up-regulated; ↓, down-regulated; CRC, colorectal cancer; GF, germ-free; ABX, antibiotics cocktail; TNF, tumor-necrosis factor; FMT, fecal microbiota transplantation; DSS, dextran sulfate sodium; NF-κB, nuclear factor kappa B; *P. anaerobius, Peptostreptococcus anaerobius*; IL-1, interleukin-1; LP, lamina propria; SI, small intestine; *T. gondii, Toxoplasma gondii*; VSL#3, probiotic mixture; CD, Crohn’s disease; IL-6, interleukin-6; UC, ulcerative colitis; LPS, lipopolysaccharide; IL-10, interleukin-10; IL-17, interleukin-17; IFNs, interferons; IFNAR, interferon-α/β receptor; *L. delbrueckii, Lactobacillus delbrueckii*; *S. thermophilus, Streptococcus thermophilus*; DMH, 1,2-dimethylhydrazine.
